# Interpretation of electrophysiological responses and generalization of findings requires knowledge of physical stimulus characteristics

**DOI:** 10.1007/s10633-021-09851-x

**Published:** 2021-09-18

**Authors:** Sven P. Heinrich

**Affiliations:** 1 Eye Center, Medical Center, University of Freiburg, Killianstr. 5, 79106 Freiburg, Germany; 2 Faculty of Medicine, University of Freiburg, Freiburg, Germany

Dear Editor,

With utmost interest, I read the article by Ura et al. [1], in which the authors compare VEP responses recorded to pattern-reversal stimuli that were presented on either a cathode-ray tube (CRT) or a liquid–crystal display (LCD) monitor. Because visual electrophysiology relies critically on precise stimulation, the topic is of great importance.

The utility of Ura et al.’s report would be further enhanced if we knew more about the actual stimulus properties. What matters, in the end, are the characteristics of the temporally variable luminance distribution on the screen, whether it is a CRT monitor or an LCD monitor. Without knowledge of the respective details, the generalization of Ura et al.’s findings to other monitor models is hardly possible.

Some basic differences in the stimulus characteristics are, of course, inherent to the technology used by the respective monitors [2]. For instance, CRT monitors produce a luminance time course that resembles a series of brief spikes even if a stimulus is “continuously” on the screen. A white-to-black change simply means that no more luminance spikes are produced. Furthermore, stimulus onset on a CRT monitor occurs progressively from top to bottom and not all at once. LCD monitors, on the other hand, usually suffer from differences in the temporal characteristics of black-to-white and white-to-black changes. This may result in brief transient changes in global (spatially averaged) luminance even when a checkerboard simply reverses [3], potentially adding an undesired flash component to a pattern onset or reversal VEP.

Many monitor models and stimulation set-ups have their own peculiarities beyond the mere fact that they use a certain display technology. As an example, Ura et al.’s CRT set-up apparently uses an interlaced signal, which is not very common for the type of application discussed here. Some modern monitors perform elaborate image processing with the aim of enhancing the viewing experience in certain consumer applications. Frequently, this results in undesired effects when the monitor is used as a stimulation device for visual electrophysiology.

Device specifications provided by the manufacturers are typically not very detailed, and usually, the monitor characteristics as relevant for comparing two monitors cannot be described comprehensively by listing a few numbers such as static luminance or contrast values. However, a simple graphical representation of the actual luminance time course on a millisecond scale will provide much of the necessary information, including rise and fall times of the luminance and steadiness of the luminance levels. This may necessitate own measurements to be performed by the authors. These could, for instance, be made for one check over a full period (two sequential reversals) of a checkerboard reversal stimulus. In order to differentiate between technical and physiological origins of temporal differences in electrophysiological responses (such as peak time delays), it is crucial to know with sufficient precision any discrepancy in stimulus timing between monitors. This also requires that appropriate measurements are made by the authors. These measurements could also be used to obtain an estimate of temporal jitter, which is rightly discussed by Ura et al.

Finally, it may also help the reader to appreciate Ura et al.’s findings if the authors would be more specific with respect to some experimental parameters that are already mentioned in the Methods section. What part of the stimulus does the luminance value of “80 cd/m^2^ or more” refer to? Was “or more” identical for both monitors, and how much more was it? What unit does the pattern size of 32 have? What was the actual refresh rate of the LCD monitor (a vertical scan frequency of 49–61 Hz is given)?

In short, comprehensive information about the physical characteristics of stimulation is an essential foundation for the interpretation of electrophysiological recordings. This is particularly true when comparing different monitor types. I wonder whether Ura et al. could possibly provide the relevant details.

## Declarations

### Statement on the welfare of animals

This letter does not contain any studies with animals performed by the author.
